# Extracellular Matrix Proteins Modulate Antimigratory and Apoptotic Effects of Doxorubicin

**DOI:** 10.1155/2012/268681

**Published:** 2012-07-01

**Authors:** Georges Said, Marie Guilbert, Hamid Morjani, Roselyne Garnotel, Pierre Jeannesson, Hassan El Btaouri

**Affiliations:** ^1^UFR Pharmacie, FRE CNRS/URCA no. 3481, Université de Reims Champagne-Ardenne, 51096 Reims, Cedex, France; ^2^UFR Medecine, FRE CNRS/URCA no. 3481, Université de Reims Champagne-Ardenne, 51096 Reims, Cedex, France; ^3^UFR Sciences, FRE CNRS/URCA no. 3481, Université de Reims Champagne-Ardenne, 51687 Reims, Cedex 2, France

## Abstract

Anticancer drug resistance is a multifactorial process that includes acquired and *de novo* drug resistances. Acquired resistance develops during treatment, while *de novo* resistance is the primary way for tumor cells to escape chemotherapy. Tumor microenvironment has been recently shown to be one of the important factors contributing to *de novo* resistance and called environment-mediated drug resistance (EMDR). Two forms of EMDR have been described: soluble factor-mediated drug resistance (SFM-DR) and cell adhesion-mediated drug resistance (CAM-DR). Anthracyclines, among the most potent chemotherapeutic agents, are widely used in clinics against hematopoietic and solid tumors. Their main mechanism of action relies on the inhibition of topoisomerase I and/or II and the induction of apoptosis. Beyond this well-known antitumor activity, it has been recently demonstrated that anthracyclines may display potent anti-invasive effects when used at subtoxic concentrations. In this paper, we will describe two particular modes of EMDR by which microenvironment may influence tumor-cell response to one of these anthracyclines, doxorubicin. The first one considers the influence of type I collagen on the antimigratory effect of doxorubicin (CAM-DR). The second considers the protection of tumor cells by thrombospondin-I against doxorubicin-induced apoptosis (SFM-DR).

## 1. Tumor Cell Microenvironment and Drug Resistance

In the last decade, the earlier point of view of tumors as a relatively homogeneous cancer cells has been totally changed into considering tumors as high complex organs. The autonomous properties of cancer cells are no longer sufficient to elucidate the multistep process of tumorogenesis. In fact, a better understanding of this process needs to take into consideration the stromal cells and the extracellular matrix (ECM) proteins that constitute the tumor microenvironment. These elements are known to contribute to the development and the expression of certain cancer hallmarks [1].

It is well documented that anticancer drug resistance represents a major obstacle for the successful treatment of various human malignancies. This process is multifactorial and can be subdivided into two broad categories: acquired and environment-mediated drug resistance (EMDR). Acquired resistance develops during treatment as a result of sequential genetic changes leading mainly to overexpression of drug transporters and alterations in drug targets [2]. Conversely, EMDR a form of *de novo* resistance allows cancer cells to tolerate the stress induced by therapies during the first exposure to anticancer drugs. It represents the primary way for tumor cells to escape the cytotoxic effect of anticancer drugs and could be therefore a potential target to overcome resistance to chemotherapy. Two forms of EMDR have been recently described; they are rapidly induced by signaling events resulting from direct cell contact with tumor microenvironment. The first, so-called the soluble factor-mediated drug resistance (SFM-DR) is induced by mediators such as cytokines, chemokines, and growth factors secreted by both tumor and stromal cells. The second, the cell adhesion-mediated drug resistance (CAM-DR) is mediated by the adhesion of tumor cell integrins to microenvironmental factors such as ECM components (collagen, fibronectin, and laminin) and ligands expressed on stromal cells especially cancer-associated fibroblasts. CAM-DR has been extensively described to confer potential resistance in leukemic and solid tumor cell lines to various chemotherapeutic agents [3].

Anthracyclines, one of the most potent classes of chemotherapeutic agents, are widely used in clinics against hematopoietic and solid tumors. Their main mechanism of action relies on the induction of cytotoxic or apoptotic effects. Indeed, in a wide panel of cancer cell lines, these drugs have been shown to trigger apoptosis via endogenous ceramide increase, mitochondrial and caspases pathways [4]. Beyond this well-known antitumor activity, it has been recently demonstrated in conventional cell culture on plastic substrate that they may display potent anti-invasive effects when used at subtoxic concentrations. Doxorubicin and related compounds such as aclacinomycin and DA-125, a doxorubicin analogue, have been shown to inhibit *in vitro* invasion of tumor cell lines originating from various solid tumors (prostate, breast, fibrosarcoma, etc.). This anti-invasive effect involves downregulation of matrix metalloproteinases (MMPs) [5], disorganization of cytoskeleton and focal adhesion contacts via inhibition of focal adhesion kinase (FAK) [6]. However, the primary pharmacological target of such an anti-invasive effect is still to be determined.

In this paper, we will describe two particular modes of EMDR by which microenvironment may influence tumor cell response to one of these anthracyclines, doxorubicin. The first considers the influence of type I collagen on the antimigratory effect of doxorubicin in HT1080 human fibrosarcoma cells (CAM-DR). The second considers the protection of human thyroid carcinoma FTC-133 cells by thrombospondin-I against doxorubicin-induced apoptosis (SFM-DR).

## 2. Influence of Type I Collagen on the ****Antimigratory Effect of Doxorubicin

### 2.1. Collagen-Based Cell Migration Models

In humans, type I collagen is the main component of ECM in connective tissues, through which tumor cells usually move to form metastasis and use as a preintravasation microenvironment [7]. Type I collagen is characterized by a triple-helical structure, two *α*_1_ and one *α*_2_ chains, which are stabilized by the formation of cross-links between and within the triple-helices, allowing it to form a fibrillar network. It mediates the biomechanical stability of connective tissue and provides a 3D scaffold to which other ECM proteins such as fibronectin and glycosaminoglycans are connected [8].

Early *in vitro* studies of cell adhesion and migration have been performed using two-dimensional (2D) rigid, planar substrates, coated with different types of ECM proteins such as type I collagen or fibronectin. Recently, these conventional cell migration models have given place to reconstituted 3D collagen matrices. These scaffolds offer both more realistic view due to the coupling of chemical and mechanical signals that take place in the real tissues and better simulation of cell response to anticancer drugs [9]. We here employed processed adult rat tail collagen at a concentration of 1.5 mg/mL known to be close to that found *in vivo* [10]. The highly fibrillar organization of this matrix therefore approximates the 3D fibrous nature of mesenchymal stroma [11]. In addition, due to its preparation without pepsinization, this native fibrillar collagen presents intact telopeptides in contrast with most of the experimentally generated collagen matrices currently using pepsin-cleaved type I collagen [12]. Telopeptides which correspond to the flanking regions of the molecules permit to form intra- and intermolecular cross-links that promote the staggering and the resilience of fibrillar collagen [13]. Such 3D models are well suited to directly analyze the migratory behavior and the morphology of individual moving cells by using time-lapse videomicroscopy. The *in situ* cell cytoskeleton organization after actin staining and the proliferative capacity following collagenase treatment can be also estimated. In addition, culturing tumor cells in 3D is a well-adapted approach for evaluating the cytotoxic and anti-invasive effects of anticancer drugs with the condition that their diffusion capacities have been previously tested through matrices. Indeed, drug penetration into matrices strongly depends on their molecular weight as demonstrated for the low diffusion rate of antibodies [14] compared to unaffected diffusion rate of doxorubicin representative of low molecular weight drugs [11].

### 2.2. Type I Collagen Protects Tumor Cells against the Antimigratory Effect of Doxorubicin

The influence of type I collagen on the antimigratory effect of doxorubicin was investigated by using the highly invasive fibrosarcoma cell line HT1080. At drug concentrations exhibiting no or limited effect on tumor cell proliferation, the ability of doxorubicin to decrease tumor cell motility was studied in 2D and 3D collagen-based systems. For this purpose, tumor cells were cultured either on 2D coatings [15] or within 3D matrices of type I collagen [16] and in parallel conventional 2D cultures were performed by seeding cells on plastic substrata. Exploring the role of doxorubicin at subtoxic concentrations permitted to exclude that the antimigratory effect of the drug could result from a nonspecific consequence of its cytotoxic effect. This is also of interest for therapeutic application since the use of doxorubicin is often limited by severe cardiocytotoxicity and other side effects [17].

On conventional plastic surfaces, the subtoxic concentrations of doxorubicin induced a marked inhibition of HT1080 cell migration as demonstrated by time-lapse videomicroscopy. Individual cell velocity is decreased but the frequency and mean length of breaks were not affected. In addition, cell trajectories were profoundly modified since cells exhibited shorter or circular paths around their starting point in accordance with cell speed inhibition. These results indicate that the antimigratory effect preferentially takes place during the locomotory and not the stationary phase of the cells. By contrast, this deleterious effect on cell velocity and trajectories was totally abolished when doxorubicin-treated cancer cells were cultured, respectively, on type I collagen-coated surfaces or inside 3D type I collagen matrices (Table 1). Such results are completely in agreement with inhibitory effects of ECM components on the cytotoxic mechanism of various anticancer drugs [18].

### 2.3. Type I Collagen Abolishes Doxorubicin Effects on the Migratory Molecular Regulators

**Figure 1:**
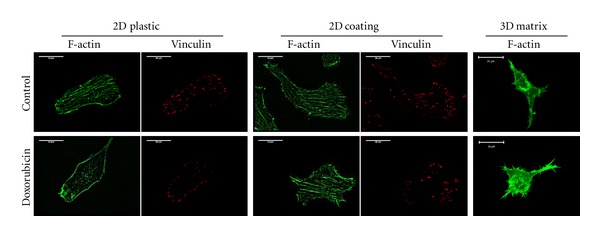
Type I collagen protects HT1080 cells against the doxorubicin-induced disorganization of cytoskeleton. After 24 h of exposure to subtoxic concentrations of doxorubicin (5 and 10 nM), cells were stained for F-actin or vinculin. (Bar = 20 *μ*m).

**Figure 2:**
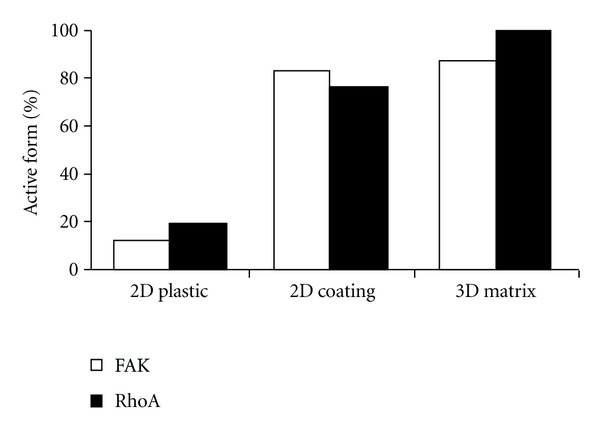
Type I collagen protects HT1080 cells from doxorubicin-induced dephosphorylation of FAK and RhoA. After 24 h of exposure to doxorubicin (5 and 10 nM), cells cultured on plastic or 2D coated type I collagen were directly lyzed, except for those cultured inside 3D matrices that were beforehand digested by collagenase P. The expression and the activation state of FAK and RhoA were quantified by western blot. *Y*-axis corresponds to the percentage ratio of active form of FAK or RhoA in doxorubicin-treated cells with respect to untreated cells.

Cell migration is a complex multistep process involved in the movement of cells from one location to another contributing therefore to cancer metastasis. The formation of new adhesions *via* the assembly of focal adhesion complexes and the remodeling of the cytoskeleton via actin stress fiber recruitment appear as key mechanisms that regulate cell motility. On plastic surfaces, doxorubicin induced a dramatic disturbance of adaptor proteins of the focal adhesion complexes such as vinculin and FAK (Figures 1 and 2). This indicates that doxorubicin acts through altering signaling complexes normally involved in the signal transmission from the ECM to the cell cytoskeleton, which could constitute the primary targets of its antimigratory effect. In presence of the drug, vinculin, an actin binding protein recruited to the *β* integrin cytoplasmic tail, is delocalized from the end of each stress fibers to the rim of the cells. In addition, the drug strongly inhibited the phosphorylation state of FAK on Tyr^397^ without modifying its expression, altering therefore FAK signaling. Concerning the cytoskeleton, doxorubicin induces complete disorganization of actin with dramatic loss of stress fibers (Figure 1). This is accompanied by a marked inhibition of the phosphorylation state of the GTPase RhoA, a key protein involved in the regulation of actin stress fiber formation. By contrast, with collagen in 2D and 3D conditions, these effects on cytoskeleton organization and on the activation state of FAK and RhoA were completely abolished (Figures 1 and 2). Taken together, these data demonstrate that type I collagen is able to protect tumor cells against the anti-invasive effect of anthracyclines.

## 3. Modulation of Chemotherapy-Induced ****Apoptosis by Extracellular Matrix Components

Several studies have progressively shown the role of the ECM in the modulation of the cell response to chemotherapy (survival/resistance) [3, 20, 21]. It is admitted now that the modulation of cell response to chemotherapy by ECM contributes to a new form of *de novo* resistance. The group of W. S. Dalton was the first to show that ECM is able to confer resistance to chemotherapy [3, 22, 23]. They demonstrated the role of fibronectin in tumor cell protection against chemotherapy via *β*1 integrin [24, 25]. This protection occurs through the activation of PI 3-kinase signaling pathway [26]. This property has been also reported for vitronectin via *α*v*β*3 and *α*v*β*5 integrin [27]. By contrast, other components of the ECM are able to sensitize tumor cells to the cytotoxic effect of anticancer drugs. TGF*β*1 is able to sensitize ovarian carcinoma cells to paclitaxel [28]. In this case, it has been clearly demonstrated that low TGF*β*1 expression could be a poor prognosis in patients [29].

Thrombospondin-1 (TSP-1) is known for playing role in the induction of apoptosis in endothelial cells via the CD36 receptor [30]. Besides this antiangiogenic effect, this protein is also able to modulate the response of tumor cells to chemotherapy via the CD47 receptor. As mentioned for TFG*β*1, TSP-1 is also able to sensitize prostate carcinoma cells to the cytotoxic effect of taxol [31, 32]. Clinical data confirmed the *in vitro* model and demonstrated that tumoral expression of TSP-1 predicts overall survival of patients with lung adenocarcinoma treated with first-line docetaxel-gemcitabine regimen [33]. Other authors have demonstrated that cisplatin was able to reverse resistance to taxol in nasopharyngeal carcinoma by upregulating TSP-1 expression [34]. Akiyamas group has reported that 5-fluorouracil (5-FU) induced TSP-1 in human colon carcinoma cells. A transcription factor, Egr-1, was also induced by 5-FU and bound to the promoter of TSP-1, enhancing its transcription and the subsequent production of TSP-1 protein. Moreover, this group presented the evidence that p38 mitogen-activated protein kinase (MAPK) plays an important role in 5-FU—induced Egr-1 transactivation [35]. Because estrogens cause progression of many breast cancers, Wu laboratory have examined whether TSP-1 is regulated by estrogen. Estradiol (E2) induced TSP-1 expression in human breast cancer cells *in vitro*. This induction was blocked by the estrogen antagonists, indicating that estrogen receptors are necessary for this effect. Furthermore, E2 caused the production of TSP-1 protein from tumor cells in an ER-*α*-dependent manner [36]. The same authors have shown that synthetic progestins also induce TSP-1 mRNA and protein in human breast cancer cells. Antiprogestin RU-486 was able to inhibit the induction of TSP-1 by progestins, suggesting also that this effect is mediated by the progesterone receptor [37].

Our group has reported recently that doxorubicin is able to induce apoptosis in thyroid carcinoma cells via ceramide *de novo* synthesis [38]. Moreover, this apoptosis is accompanied by a downregulation of TSP-1 expression at mRNA and protein levels [39]. The addition of TSP-1 protects the cells against doxorubicin-induced apoptosis [39]. The antiapoptotic role of TSP-1 involves its C-terminal part that interacts with the membrane receptor CD47. More recently, we have shown that both doxorubicin was able to activate JNK/ATF-2 pathway to downregulate TSP-1 expression and to modulate apoptosis [40]. The role of TSP-1 in the protection of thyroid carcinoma cells will be discussed in more detail in this second part of the paper.

### 3.1. Structural Organization and Role of TSP-1

TSP-1 is an ECM glycoprotein first discovered in activated platelets [41]. TSP-1 has been the first identified member of the thrombospondin family in 1971 [42] since this protein was released in response to thrombin in activated platelets and participated to the formation of the fibrin clot [43]. Different data are now available about the role of TSP-1 in cancer and support the hypothesis of important functions for TSP-1 in tumor growth and metastasis. However, conflicting results led to consider TSP-1 either as a tumor suppressor or as a tumor promoter [44].

The cascade of signal transduction following interaction between the cell and ECM components constitutes an interesting way of investigation since ECM proteins are recognized as important regulators of cell growth and function. TSP-1 presents multiple structural domains and putative ligand binding sites including integrins, CD36 [45], CD47, low-density lipoprotein (LDL) receptor-related-protein and proteoglycans [46]. This diversity implies that TSP-1 can interact at the same time with one or more receptors in a cell type. Otherwise, the cell response to TSP-1 can differ according to the respective levels of receptors expression and leads to opposite responses depending on the physiopathological situation.

TSP-1 is expressed on the cell surface during physiological events. A variety of normal cells, including endothelial cells, fibroblasts, adipocytes, smooth muscle cells, monocytes, macrophages, and transformed cells such as malignant glioma cells to eliminate secrete TSP-1 [47, 48].

**Figure 3:**
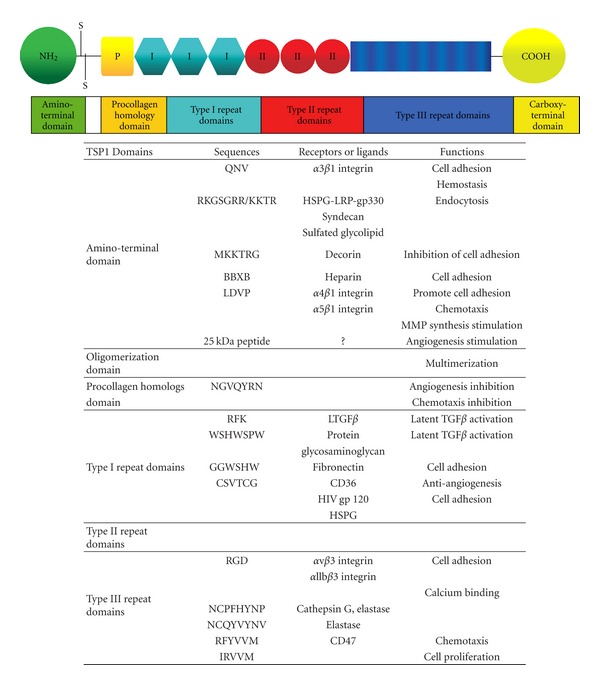
Schematic representation of the multimodular structure and functions of a single subunit of the TSP-1. TSP-1 presents several distinct domains with specific biological properties. In the table are indicated the known peptide sequences, the corresponding receptors or ligands, and the associated biological activities [19].

TSP-1 contains a N-terminal globular domain that binds heparin, type I, type II, and type III repeats, and a terminal globular domain. The structure of TSP-1 is schematically shown in Figure 1. The NH_2_-terminal of TSP-1 interacts with low-density lipoprotein receptor-related protein (LRP1) [49], heparin sulfate proteoglycans and a number of integrins that have an important function in angiogenesis, chemotaxis adhesion, and cell motility [50]. All five members of the TSP family have the repeat domains type II and III, but only TSP-1 and TSP-2 contain the type I repeats [51]. Type I repeats, also called thrombospondin structural homology repeats (TSRs), inhibit angiogenesis by activating CD36 and inducing apoptosis in endothelial cells [52] (Figure 3). 

The COOH-terminal domain of TSP-1 binds to CD47, also known as integrin-associated protein [53]. This domain also interacts with integrins such as *β*1 and *β*6 integrins and actively binds to proteoglycans allowing cell adhesion and spreading [50]. These and other interactions significantly affect angiogenesis, cell proliferation, and immune responses (Figure 3).

### 3.2. TSP-1 and Chemotherapy

Previous studies have developed the concept that the levels of TSP-1 could be directly correlated with the resistance and aggressiveness of the thyroid cancer. Recent data showed that FTC-238 thyroid cells, exhibiting a higher endogenous TSP-1 level than FTC-133, were the less sensitive to doxorubicin treatment [54]. In fact, TSP-1 was previously linked to disease recurrence and decreased patient survival [55, 56]. Some reports investigated TSP-1 expression level in clinical thyroid cancer cases. One study showed no significant difference in mean TSP-1 mRNA expression in *in vivo* thyroid cancers in comparison to normal specimens [57]. However, another study demonstrated that TSP-1 expression was reduced in correlation with the increasing aggressiveness of different thyroid lesions [58]. Another report evaluating 75 papillary thyroid cancers, demonstrated that TSP-1 expression was inversely correlated with invasiveness [59]. Indeed, patients with TSP-1-negative tumors appeared to present a poor prognosis in colon cancer [60], and overexpression of TSP-1 in mice lacking endogenous TSP-1 was reported to suppress tumor growth [61].

The effects of TSP-1 have been studied in many preclinical tumor models, and mimetic peptides are being tested in cancer clinical trials. Indeed, the interaction of TSP-1 with the nitric oxide pathway seems to be involved in the anti-angiogenic mechanisms mediated by TSP-1 TSR-derived peptide in cancers [62, 63], and it might also explain the anti-inflammatory effects of this peptide in the colitis model [64]. Studies of prostate cancers indicate that the combined decrease of NF-*κ*B and increase of TSP-1, modulated by the expression of the androgen receptor, exert antitumor effects [65]. The TSP-1-derived peptide angiocidin has antitumor effects and induces the differentiation of monocytes to macrophages by activating the NF-*κ*B pathway [66].

### 3.3. Doxorubicin Induces Apoptosis and Downregulates TSP-1 Expression in Human Thyroid Carcinoma FTC-133 Cells

We have reported that doxorubicin, an inhibitor of topoisomerase II, led to elevated cytotoxic events associated with ceramide generation and correlated with TSP-1 down regulation, mainly occurring at the transcriptional level and an induced apoptosis in human thyroid carcinoma FTC-133 cells [38, 39]. 

Ceramide generation can occur through hydrolysis of sphingomyelin, that is, catalyzed by acid or neutral sphingomyelinase or through *de novo* synthesis starting with serine and palmitate condensation [67–70].

**Figure 4:**
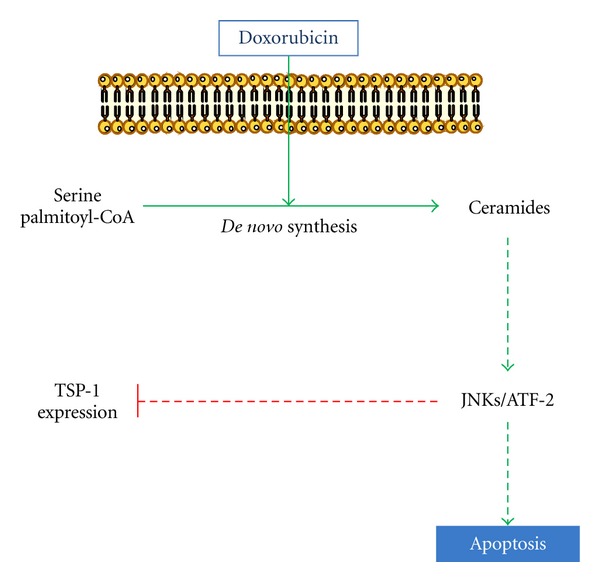
Schematic representation of signaling pathway mediated by doxorubicin in inhibition of TSP-1 expression and induction apoptosis.

In our study, we demonstrated that neither acid nor neutral sphingomyelinase-dependent activities varied upon doxorubicin treatment in FTC-133 cells. Doxorubicin enhanced ceramide production mainly via the *de novo* synthesis pathway. This pathway usually results in a prolonged ceramide elevation and was responsible for the drug-induced malignant cell apoptosis through a caspase-3-dependent pathway and a decrease of TSP-1 amount [38] (Figure 4).

C-Jun N-terminal kinases (JNKs) are multifunctional signaling networks that influence cell proliferation, differentiation, apoptosis, and responses to stress playing a pivotal role in the signal transduction from different stimuli [71]. We confirmed that the JNK pathway plays a central role in doxorubicin-induced downregulation of TSP-1 and apoptosis in FTC-133 cells. Moreover, doxorubicin induced JNK phosphorylation through *de novo* ceramide synthesis and chemical inhibition of either ceramide production or JNK activation prevent doxorubicin effects on TSP-1 expression and cell apoptosis. Other reports have also clearly established the role of JNK in X-ray, doxorubicin and TRAIL-induced apoptosis in U937 cells, rat hepatoma cell line, mouse lymphocytic leukemia cells and squamous cell carcinoma, respectively [72–75]. Moreover, JNK inhibition abrogated estradiol-induced suppression of TSP-1 synthesis in endothelial cells [76]. 

JNK phosphorylates the transcription factors c-Jun, ATF-2, Elk-1, p53, and c-Myc, as well as antiapoptotic Bcl2 family members [77]. It has been proposed that JNK-induced ATF-2 phosphorylation might control apoptosis in response to stresses such as UV light, osmotic stress, hypoxia, and inflammatory cytokines [78, 79]. 

Our data demonstrated that ATF-2 was phosphorylated in response to these agents. This phosphorylation was inhibited by SP600125 indicating that JNK controlled ATF-2 activation. Moreover, ATF-2 silencing abolished doxorubicin effects. Other reports have also demonstrated that JNK inhibition led to a decrease of active caspase-3 and ATF-2 phosphorylation which correlated with a decrease in the number of apoptotic cells [80]. In summary, our data put in evidence that JNK/ATF-2 is activated by doxorubicin via* de novo* ceramide leading to TSP-1 downregulation and consequently apoptosis in FTC-133 cell (Figure 4).

### 3.4. Anti-apoptotic Effect of TSP-1 in FTC-133 Cells

**Figure 5:**
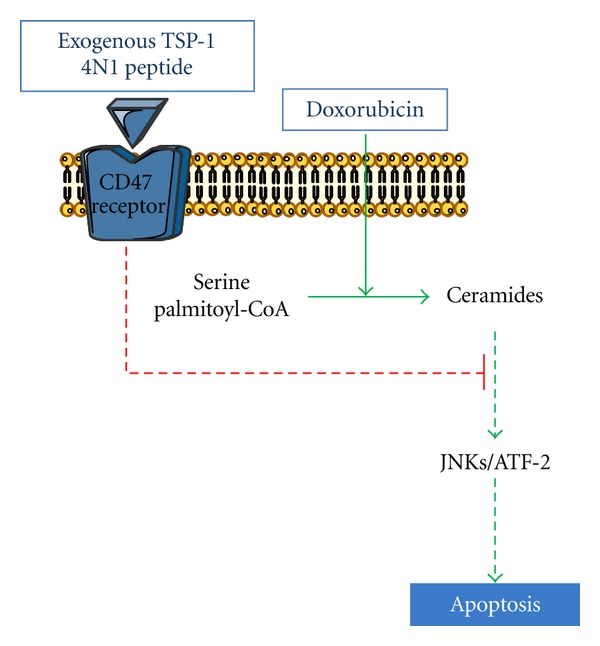
Schematic representation of protective effect of TSP-1 C-terminal-derived peptide 4N1 against doxorubicin-induced apoptosis in FTC-133 thyroid cells.

The correlation between TSP-1 downregulation and apoptosis was confirmed by other investigation underlined that overexpression of the PTEN tumor-suppressor gene leading to induction of thyroid carcinoma apoptosis was also correlated with a significant downregulation of TSP-1 expression at both RNA and protein levels [81]. These results led us to investigate a possible antiapoptotic role for TSP-1 in FTC-133 cells. Our results shed new light on the antiapoptotic properties of TSP-1 protein in thyroid cancer. We provided evidences that doxorubicin-induced apoptosis was significantly decreased in the presence of CD47/IAP-binding peptide 4N1, a peptide derived from the COOH-terminal domain of TSP-1 [39]. These results were confirmed by anti-CD47 antibody that blocked 4N1 protective effect [39]. These findings suggest that induction of apoptosis by doxorubicin in FTC-133 cells is dependent on the downregulation of TSP-1 expression shedding light on a potential role for TSP-1 in cell response to chemotherapy (Figure 5).

Recent studies described that some cancers may develop the ability to counterbalance their own secretion of pro- or antitumoral factors [82–84] which could explain the apparent conflicting results. Some tumor cells were shown to bypass the expected inhibitory effect of TSP-1. For example, human breast cancer cells could override the antiangiogenic effects of TSP-1 *in vivo* by increasing vascular endothelial growth factor (VEGF) expression [85]. In FTC-133 cells, a continuous inhibition of TSP-1 under drug treatment was observed and addition of TSP-1 or its derived peptide 4N1 in doxorubicin-treated cells exhibited an unexpected antiapoptotic effect. Whether other growth factors are implicated remains to be demonstrated. Altogether, according to the cellular environment specificity, TSP-1 could be associated with a worse prognosis, and therefore, considered as an interesting marker for patient survival.

Finally, these data shed light on one component of the drug-resistance phenotype in thyroid tumoral diseases. They support the idea that TSP-1 could be helpful for predicting recurrence and survival outcome in patients affected by such pathologies. Understanding how biological factors such as TSP-1 are capable of modulating tumor-cell response to chemotherapy will be of great interest to enhance therapeutic response and to identify efficient clinical chemotherapeutic protocols.

## 4. Concluding Remarks

ECM proteins were demonstrated to protect tumor cells against the antimigratory and apoptotic effects of antitumor drugs. These data support the crucial role of the tumor microenvironment in the failure of clinical response to chemotherapeutic agents and the emergence of EMDR. In addition, they suggest that tumor cell/ECM interactions should be taken into account in the development of new agents specifically targeting tumor cell proliferation and motility in order to prevent metastasis. More generally, this paper highlights that traditional cell culture models are insufficiently representative of solid tumors, recently considered as complex organs [1]. It suggests that the remodeling of tumor microenvironment could represent an innovative approach [86] to improve therapeutic efficacy of conventional anticancer drugs previously thought to be ineffective.

## Figures and Tables

**Table 1:** Type I collagen protects HT1080 cells against the antimigratory effect of doxorubicin. The seeded cells on plastic, on 2D coating or inside 3D matrices, were exposed to subtoxic concentrations of doxorubicin (5 and 10 nM) for 24 h. Cell motility was examined for the last 12 h using time-lapse videomicroscopy as described before [15, 16].

	Migration speed (*μ*m/h)		
	2D plastic	2D coating	3D matrix
Control	15.6 ± 1.4	19.0 ± 0.7	24.8 ± 0.9
Doxorubicin	8.3 ± 0.8	23.0 ± 1.1	25.7 ± 1.5
